# Dynamic and subtype-specific interactions between tumour burden and prognosis in breast cancer

**DOI:** 10.1038/s41598-020-72033-3

**Published:** 2020-09-22

**Authors:** S. B. Lee, H.-K. Kim, Y. Choi, Y. W. Ju, H.-B. Lee, W. Han, D.-Y. Noh, B. H. Son, S. H. Ahn, K. S. Kim, S. J. Nam, E.‑K. Kim, H. Y. Park, W.-C. Park, J. W. Lee, H.-G. Moon

**Affiliations:** 1 Division of Breast Surgery, Department of Surgery, Asan Medical Centre, University of Ulsan College of Medicine, 88 Olympic-ro 43-gil, Songpa-gu, Seoul, 05505 Korea; 2 Department of Surgery, Seoul National University College of Medicine, 28 Yongon-dong, Chongno-gu, Seoul, 110-744 Korea; 3 Division of Breast and Endocrine Surgery, Department of Surgery, Korea University Anam Hospital, Korea University College of Medicine, Seoul, Korea; 4 Medical Research Collaborating Centre, Seoul National University Hospital, Seoul, Korea; 5 Breast-Thyroid Centre, Ulsan City Hospital, Ulsan, Korea; 6 Division of Breast Surgery, Department of Surgery, Samsung Medical Centre, Seoul, Korea; 7 Department of Surgery, Seoul National University Bundang Hospital, Seongnam, Korea; 8 Department of Surgery, School of Medicine, Kyungpook National University, Daegu, Korea; 9 Department of Surgery, Seoul St. Mary’s Hospital, College of Medicine, The Catholic University of Korea, Seoul, Korea

**Keywords:** Cancer, Breast cancer

## Abstract

We investigated the relationship between the prognostic importance of anatomic tumour burden and subtypes of breast cancer using data from the Korean Breast Cancer Registry Database. In HR+/HER2+ and HR−/HER2−tumours, an increase in T stage profoundly increased the hazard of death, while the presence of lymph node metastasis was more important in HR+/HER2+ and HR−/HER2+ tumours among 131,178 patients with stage I–III breast cancer. The patterns of increasing mortality risk and tumour growth (per centimetre) and metastatic nodes (per node) were examined in 67,038 patients with a tumour diameter ≤ 7 cm and < 8 metastatic nodes. HR+/HER2− and HR−/HER2− tumours showed a persistent increase in mortality risk with an increase in tumour diameter, while the effect was modest in HER2+ tumours. Conversely, an increased number of metastatic nodes was accompanied by a persistently increased risk in HR−/HER2+ tumours, while the effect was minimal for HR−/HER2− tumours with > 3 or 4 nodes. The interactions between the prognostic significance of anatomic tumour burden and subtypes were significant. The prognostic relevance of the anatomic tumour burden was non-linear and highly dependent on the subtypes of breast cancer.

## Introduction

Breast cancer mortality rate has been declining in Western countries due to advancements in early detection and systemic therapies^1^; however, a substantial proportion of patients with early breast cancer eventually develop disease recurrence. A recent study reported that more than 30% of patients with operable breast cancer developed disease recurrence during a 24-year long follow-up period^2^. The accurate prediction of outcomes of individual patients helps both patients and clinicians during the decision-making process for therapy, and remains the cornerstone of personalised medicine.

Traditionally, the prediction of recurrence of solid tumours has been mainly based on information regarding tumour size and nodal metastasis. In addition to this anatomic staging information, clinicians have long considered the biologic characteristics of the tumour, such as the molecular subtype and histologic grade of breast cancer, as essential factors influencing survival outcomes. The model for predicting breast cancer survival by integrating anatomic staging, biologic characteristic and treatment data was developed using National Cancer Data Base^3^. Recently, the 8th American Joint Committee on Cancer (AJCC) staging system incorporated biologic factors, including tumour grade, estrogen receptor (ER) status, progesterone receptor status, and human epidermal growth factor receptor 2 (HER2) status, into the traditional anatomic staging factors to develop the prognostic staging system^4^.

The validity of the 8th AJCC staging system originates from studies that examined the usefulness of the novel scoring system (Bioscore), which includes both anatomic and biologic information, in large cohorts of patients with breast cancer^5,6^. This scoring system was developed by calculating the degree of contribution for each prognostic factor (i.e., tumour size, node status, and ER status) based on the results of multivariate survival analyses. Since the analyses were not stratified by molecular subtype, the scoring system assumes that changes in tumour size and nodal status result in similar prognostic effects among different tumour subtypes. However, it remains unclear whether the anatomic prognostic factors yield linear effects on the survival or if there are potential interactions between the prognostic effect of anatomic factors and biologic characteristics. Some studies have shown possible interactions between the prognostic relevance of the anatomic staging factors and biologic characteristics of tumours in breast cancer^7–9^.

This study aimed to determine whether the degree of prognostic importance for tumour size and nodal status (anatomic burden) is influenced by the subtype (biologic factors) using data from a large cohort of Korean patients with breast cancer. Further, we assessed the patterns of incremental risks of mortality according to changes in tumour size (cm) and nodal status (number of metastatic nodes) in different subtypes of breast cancer to determine whether the anatomic burdens exhibit linear prognostic significance.

## Patients and methods

This study was approved by the institutional review board of Seoul National University Hospital.

### The Korean breast cancer society registry

The Korean Breast Cancer Society Registry (KBCSR) is a nationwide, prospectively maintained registry program for newly diagnosed patients with breast cancer in Korea^10^. As of 2019, 131 teaching hospitals in Korea are contributing to the registry, and a total of 192,232 patients are registered in the KBCSR database. The KBCSR database contains data regarding sex, age, type of operation, date of diagnosis, cancer stage based on the AJCC classification system, histological grade, nuclear grade, immunohistochemical status of ER, PR, and HER2, and treatment methods. The survival data was provided by the National Statistical Office, Korea which is more reliable for the overall survival and less accurate for the breast cancer-specific survival; therefore, we assessed the overall survival, not breast cancer-specific survival in this study. The KBCSR database does not contain data regarding the status of breast cancer recurrence or the date of recurrence.

In this study, patients were included if they met the following criteria: female sex, age of diagnosis between 18 and 70 years, unilateral breast cancer, stages T1–T4 disease, and N1–N3 disease. We excluded patients with distant metastasis at diagnosis, metastasis to the internal mammary or supraclavicular lymph nodes, and unknown tumour size or pathology results. To determine the detailed changes in prognostic patterns according to tumour size and lymph node status, we additionally identified patients with a tumour diameter of ≤ 7 cm in its widest dimension and who had ≤ 7 metastatic lymph nodes. The cutoff values for tumour size or the number of metastatic nodes were determined by the availability of an adequate number of patients in each size or node group that provided reliable statistical results (Supplementary Table S1). Hormone receptor (HR) positivity was defined by immunohistochemistry (IHC) for ER and/or PR (at least 1 percent of invasive tumour cells) according to American Society of Clinical Oncology and College of American Pathologists guidelines. HER2 positivity was defined by staining more than 30 percent of invasive tumour cells (IHC score 3+) or the presence of HER2 gene amplification by in situ hybridization. Tumours were classified into four different subtypes based on the HR status and HER2 expression status: (1) HR+/HER2− subtypes, (2) HR+/HER2+ subtypes, (3) HR−/HER2+ subtypes, or (4) HR−/HER2− subtypes.

### Statistical analyses

We assessed the overall survival of patients with breast cancer in the KBCSR database using the Kaplan–Meier method and the Cox proportional hazards model. Survival curves were generated using the Kaplan–Meier method and compared across groups using the log-rank test. For the univariate and multivariate analyses, a Cox proportional hazards model was used to estimate the unadjusted hazard ratio and the adjusted hazard ratio for significance, respectively. To address the potential interaction between the subtypes and the degree of prognostic contribution of tumour size or lymph node metastasis, the interaction terms were added and tested using a Cox proportional hazards model. All statistical analyses were performed using SPSS version 20.0 for Windows (SPSS Inc., Chicago, IL, USA).

## Results

### Differences in the prognostic impact of T and N staging across subtypes

From the KBCSR Database, we were able to identify 131,178 patients diagnosed with stage I to III invasive breast carcinoma who were treated between 1980 and 2014. The demographic and clinical characteristics of the included patients are listed in Supplementary Table S2. As expected, the TNM anatomic staging effectively predicted survival outcome among these patients (Supplementary Fig. S1). In the univariate and multivariate analyses of factors influencing the survival of these patients, well-known prognostic factors such as tumour size, nodal status, histologic grade, and subtype remained as significant prognostic factors in the present cohort (Supplementary Tables S3 and S4).

**Figure 1:**
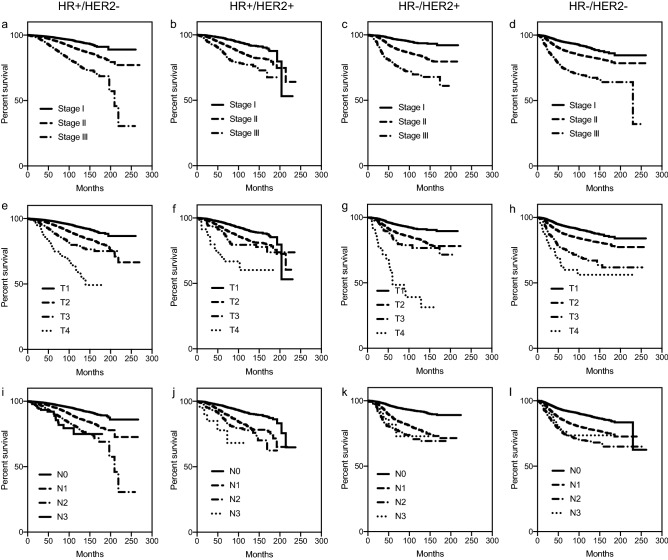
Prognostic significance of TNM staging information in different subtypes of breast cancer. Kaplan–Meier curve of overall survival according to stage (**a**–**d**), T stage (**e**–**h**), and N stage (**i**–**l**) stratified by tumour subtypes (HR+/HER2−, HR+/HER2+, HR−/HER2+, and HR−/HER2−).

**Figure 2:**
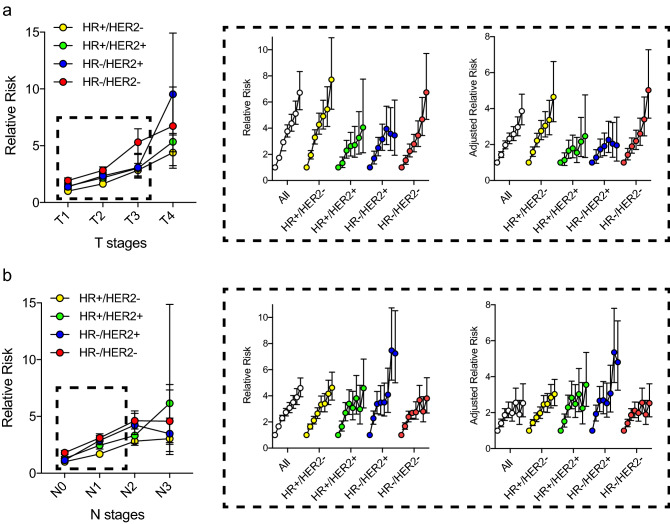
Adjusted mortality risks according to tumour status (**a**) and nodal status (**b**). The relative risk of mortality among 131,178 breast cancer patients was assessed according to different T and N stages of various subtypes when compared with T1 or N0 HR+/HER2− tumours as the reference group. Subgroup analysis of 67,038 patients with tumour diameter ≤ 7 cm in the widest dimension and < 8 metastatic nodes (dotted line area) was performed to determine the degree of prognostic importance as tumours increased in diameter (per cm) or disseminated to one additional axillary lymph node.

However, when the patients were classified according to the hormone receptor and HER2-overexpression status, we observed that the degree of the prognostic impact of TNM staging varied according to the subtypes (Fig. 1). For example, when stage T3 tumours were compared with stage T2 tumours, the HR+/HER2− and HR−/HER2− subtypes showed clear differences in survival, while HR+/HER2+ and HR−/HER2+ subtypes showed minimal differences. Also, the survival difference between patients with N0 and N1 tumours was most evident in HR−/HER2+ subtypes compared with that in other subtypes. To elucidate the subtype-specific prognostic effect of different T and N stages, we performed a Cox regression analysis with adjustment for other prognostic factors. Figure 2 and Supplementary Table S5 show the adjusted mortality risks according to different T and N stages of various subtypes when compared with T1 or N0 HR+/HER2− tumours as the reference group. Consistent with the Kaplan–Meier survival curves, we observed that the effects of T and N stages on survival differed among various subtypes. These findings suggest that tumour size and nodal status may exert subtype-specific, dynamic prognostic effects in patients with breast cancer.

### Patterns of mortality risk among patients with different tumour size and nodal status

To obtain more detailed information on the relationship between the tumour size or nodal status and their prognostic implication in patients with early and/or locally advanced breast cancer, we identified 67,038 patients with a tumour diameter of ≤ 7 cm in the widest dimension and who had less than 8 metastatic lymph nodes. Using this dataset, we were able to determine the degree of prognostic importance as tumours increased in diameter (per cm) or as they disseminated to one additional axillary lymph node during the early phase of tumour progression and its relationship with different subtypes. As shown in Fig. 2, different subtypes of breast cancer showed distinct characteristics in terms of the prognostic effects of tumour size and nodal status. For HR+/HER2− tumours and HR−/HER2− tumours, an increase in tumour size was accompanied by a gradual and persistent increase in the mortality risk. However, the HR+/HER2+ and HR−/HER2+ tumours showed a modest or minimal increase in the mortality risk when the tumour diameter exceeded 2 or 3 cm after adjusting for other prognostic factors. Regarding the number of metastatic lymph nodes, HR−/HER2+ tumours showed a dramatic increase in the mortality risk as more lymph nodes were involved. In contrast, an increase in the number of metastatic lymph nodes in HR−/HER2− tumours had limited effects on survival outcome, especially when more than 3 or 4 nodes were involved. To adjust HER2 status and the effect of trastuzumab used since 2009 in Korea, we performed additional subgroup analysis of 28,213 patients from 2009 to 2014. The patterns of relative risk in mortality showed similar findings when compared to those of the study group of 67,038 patients (Supplementary Fig. S2).

To address the statistical significance of the potential interaction between subtypes and the degree of prognostic contribution by tumour size or lymph node metastasis, we tested the interaction terms in a Cox proportional hazards model and estimated the subtype-specific effects of tumour size and number of involved lymph nodes (Table 1). The data showed that each unit increase in tumour diameter (in cm) conferred a 28% and 23% increase in the hazard of death for HR+/HER2− and HR−/HER2− tumours, respectively. HR+/HER2+ and HR−/HER2+ tumours showed a 15–18% increase in the hazard of death. In contrast, an additional increase in the number of metastatic lymph nodes resulted in a 29% and 19% increase in the mortality risk among HR−/HER2+ and HR−/HER2− tumours, respectively. There were statistically significant differences among subtypes regarding the degree of increase in mortality risk by tumour size and lymph node metastasis status.

**Table 1:** Interactions between the prognostic effects of anatomic stages and subtypes.

	HR+/HER2−		HR+/HER2+		HR−/HER2+		HR−/HER2−		p-value^d^
	HR (95% CI)	p-value^c^	HR (95% CI)	p-value^c^	HR (95% CI)	p-value^c^	HR (95% CI)	p-value^c^	
Tumour size^a^	1.28 (1.24, 1.32)	ref	1.18 (1.11, 1.25)	0.0152	1.15 (1.09, 1.21)	0.0009	1.23 (1.19, 1.28)	0.1779	0.0031
Involved node number^b^	1.21 (1.18, 1.24)	0.0013	1.23 (1.18, 1.28)	0.0567	1.29 (1.25, 1.34)	ref	1.19 (1.16, 1.23)	0.0004	0.0032

*HG* histology grade, *LVI* lymphovascular invasion, *RT* radiation therapy, *CT* chemotherapy.

^a^Adjusted by age, HG, LVI, RT, CT, involved node number.

^b^Adjusted by age, HG, LVI, RT, CT, tumour size.

^c^Significance of HR difference versus 'ref'.

^d^Significance of HR difference across the surrogate molecular subtype.

## Discussion

The molecular subtypes of breast cancer substantially influence the survival outcomes and sites of recurrence^11–14^. We hypothesised that the clinical relevance of anatomic tumour burden would also be affected by the subtypes of breast cancer. Although previous studies also examined the degree of the prognostic effect of a small increase in tumour size or the number of metastatic lymph nodes in breast cancer, these analyses often failed to stratify patients according to subtypes^15,16^. In the present study, we showed that the degree of prognostic significance of tumour size and nodal status varied among different subtypes based on our analysis of 131,178 patients with breast cancer registered in a nationwide registry program. Further, our data suggest that the prognostic implication of tumour size and lymph node metastasis differ along with the various stages of tumour progression within the same subtype.

Additionally, we assessed the data of 67,038 patients with breast cancer with tumour diameter ≤ 7 cm and who had ≤ 7 metastatic nodes. This analysis showed that the added prognostic effect of anatomic tumour burden, as assessed via the tumour size or the number of metastatic lymph nodes was dynamic rather than linear, and the effect was strongly determined by the subtype of breast cancer. For each unit increase in tumour size (in cm), HR+/HER2− and HR−/HER2− tumours showed a relatively higher increase in mortality risk and the increasing pattern of risk persisted until the tumours reached a diameter of 7 cm. In contrast, HR−/HER2+ tumours showed the greatest increase in mortality risk accompanied by additional lymph node metastasis. Among HR+/HER2− and HR−/HER2− tumours, an increase in the number of metastatic lymph nodes was associated with a significantly lower increase in mortality risk during the N1 stage. When the number of metastatic nodes exceeded 3 or 4, there was a minimal increase in mortality risk, particularly among patients with HR−/HER2− tumours.

Since there have been changes in treatment strategies during the study period (1980–2014), the whole population included in the study is heterogeneous and cannot be considered reflecting the contemporary population. Therefore, as adjuvant trastuzumab became standard of care in 2009 in Korea, we performed a subgroup analysis of 28,213 patients from 2009 to 2014 to adjust HER2 status and the effect of trastuzumab. Treatment with trastuzumab improved the prognosis of HER2+ tumours; however, interestingly, similar mortality risk patterns were seen in the group with and without trastuzumab. Furthermore, during the period, there has been progress in radiation therapy, and it is well known that radiation therapy after breast-conserving surgery and mastectomy reduces the risk of recurrence as well as the risk of mortality of breast cancer. Indeed, our data also demonstrated the benefit of radiation therapy in a large dataset of Korean breast cancer patients which is consistent with previous studies^17–20^.

There are conflicting pieces of evidence regarding the prognostic importance of tumour size and lymph node metastasis in patients with triple-negative breast cancer. Foulkes et al.^9^ reported a non-significant association between tumour size and mortality risk among 196 patients with basal breast cancer when the tumours were classified according to cutoff diameter values of 1.5 cm and 2.0 cm. In contrast, Albergaria et al.^21^ and Rakha et al.^22^ reported that tumour size is still an important prognostic factor in triple-negative breast cancer based on their review of 155 and 282 cases, respectively. The findings of the present study which included 12,691 patients with triple-negative breast cancer with tumour diameter ≤ 7 cm clearly corroborate the latter claim that tumour size is an important prognostic indicator of triple-negative breast cancer. Regarding the importance of lymph node metastasis, our results corroborate those of the report of Hernandez-Aya et al.,^7^ which showed that once lymph node metastasis occurs, the number of positive lymph nodes had a minimal effect on prognosis in their series of 1,711 patients with triple-negative breast cancer. The minimal prognostic impact of nodal status in triple-negative breast cancer can be explained by the fact that triple-negative breast cancer grows fast and spreads hematogenously rather than through the lymphatics, leading to early visceral metastasis^14,23,24^. The importance of hematogenous spread in triple-negative tumours is further supported by studies that reported increased vascular proliferation and density in this subtype^25,26^, which may lead to increased recurrence^27^.

In this study, we found a dramatic increase in mortality risk with an increasing number of involved lymph nodes in patients with HR−/HER2+ tumours. It has been repeatedly shown that patients with HER2+ breast cancer have a higher incidence of lymph node metastasis than patients with other subtypes^28–31^. Furthermore, the degree of lymphatic vessel density has been shown to be significantly associated with subtypes of breast cancer with the HER2 subtype showing the highest density^32^. HER2 signalling in breast cancer cells is critical in heregulin β1-induced up-regulation of vascular endothelial growth factor-C, an important regulator of tumour lymphangiogenesis^33^, and the HER2 protein expression level is significantly associated with vascular endothelial growth factor-C expression and tumour lymphangiogenesis in human breast cancer tissues^34^. The findings of these studies, along with those of the present study, suggest that the lymphatic route of metastasis is of clinical importance in patients with HER2+ breast cancer. In addition, it was revealed through the subgroup analysis that the biologic behaviour of HER+ tumours persisted even after application of trastuzumab.

This study has several limitations. As mentioned earlier, during the study period of over 30 years (1980–2014), there were changes in treatment policies and guidelines. For example, during the period, trastuzumab was incorporated as a key adjuvant treatment for HER2-positive breast cancer^35^ and several improvements were made in the standard adjuvant endocrine treatment for HR + tumours^36^. Moreover, we could not determine the subtype-specific interactions between anatomic tumour burden and sites of metastasis since the KBCSR database did not contain information on tumour recurrence. As it is well known that the patterns of distant metastasis vary among different breast cancer subtypes, it would be interesting to investigate the association between tumour burden and patterns of distant failure in the future.

## Conclusions

In conclusion, our results demonstrate that the prognostic implication of tumour growth and spread, as assessed via the increase in tumour size and number of metastatic nodes, shows significant association with the surrogate molecular subtypes of breast cancer. Additionally, our results suggest that the association between prognostic impact and anatomic tumour burden is dynamic rather than linear within a specific subtype.

## Supplementary information

Supplementary file1

Supplementary file2

Supplementary file3

## Data Availability

The datasets generated during and/or analyzed during the current study are available from the corresponding author on reasonable request.
