# Supplementary material for: Dynamic and subtype-specific interactions between tumour burden and prognosis in breast cancer
Source: Sci Rep. 2020 Sep 22;10:15445. doi: 10.1038/s41598-020-72033-3 (PMC7508816; doi:10.1038/s41598-020-72033-3)
Supplement: Supplementary file 3 — Supplementary file3 [file 41598_2020_72033_MOESM3_ESM.docx]

**Supplementary Information**

**Dynamic and Subtype-Specific Interactions between Tumour Burden and Prognosis in Breast Cancer**

S.B. Lee, MD, PhD ^a#^ , H-K. Kim, MD, PhD ^b,c#^, Y. Choi, PhD ^d^, Y.W. Ju, MD^b^, H-B. Lee, MD^b^, W. Han, MD, PhD ^b^, D-Y. Noh, MD, PhD ^b^, B.H. Son, MD, PhD ^a^, S.H. Ahn, MD, PhD ^a^, K.S. Kim, MD, PhD ^e^, S.J. Nam, MD, PhD ^f^, E‑K Kim, MD, PhD ^g^, H.Y. Park, MD, PhD ^h^, W-C. Park, MD, PhD ^i^, J.W. Lee, MD, PhD ^a*^, H-G. Moon, MD, PhD ^b*^

^a^Division of Breast Surgery, Department of Surgery, Asan Medical Centre, University of Ulsan College of Medicine, Seoul, Korea;

^b^Department of Surgery, Seoul National University College of Medicine, Seoul, Korea;

^c^Division of Breast and Endocrine Surgery, Department of Surgery, Korea University Anam Hospital, Korea University College of Medicine, Seoul, Korea;

^d^Medical Research Collaborating Centre, Seoul National University Hospital, Seoul, Korea;

^e^Breast-Thyroid Centre, Ulsan City Hospital, Ulsan, Korea;

^f^Division of Breast Surgery, Department of Surgery, Samsung Medical Centre, Seoul, Korea;

^g^Department of Surgery, Seoul National University Bundang Hospital, Seongnam, Korea;

^h^Department of Surgery, School of Medicine, Kyungpook National University, Daegu, Korea;

^i^Department of Surgery, Seoul St. Mary’s Hospital, College of Medicine, The Catholic University of Korea, Seoul, Korea

^#^S.B. Lee and H-K. Kim contributed equally to this study as co-first authors.

**Supplementary Fig S1.** Prognostic significance of TNM staging information in 131,178 breast cancer patients.

**Supplementary Fig S2.** Adjusted mortality risks according to tumour status (a) and nodal status (b) of subgroup of 63,281 patients who were treated after 2009 when the trastuzumab use was approved in Korea. The relative risk of mortality was assessed according to different T and N stages of various subtypes when compared with T1 or N0 HR+/HER2- tumours as the reference group. Subgroup analysis of 28,213 patients with tumour diameter ≤7 cm in the widest dimension and ≤7 metastatic nodes (dotted line area) was performed to determine the degree of prognostic importance as tumours increased in diameter (per cm) or disseminated to one additional axillary lymph node.

Supplementary Table S1. The number of patients for each subdivision per unit (cm) increase in tumour size or for each additional axillary lymph node involved

|  | No. of patients | % |
| --- | --- | --- |
| Tumour size, cm |  |  |
| 0–1.0 | 27,358 | 20.9 |
| 1.1–2.0 | 42,862 | 32.7 |
| 2.1–3.0 | 28,871 | 22.0 |
| 3.1–4.0 | 10,547 | 8.0 |
| 4.1–5.0 | 4,322 | 3.3 |
| 5.1–6.0 | 2,483 | 1.9 |
| 6.1–7.0 | 1,235 | 0.9 |
| 7.1–8.0 | 776 | 0.6 |
| 8.1–9.0 | 413 | 0.3 |
| 9.1–10.0 | 360 | 0.3 |
| >10 | 582 | 0.4 |
| Unknown | 11,369 | 8.7 |
| Number of involved lymph node |  |  |
| 0 | 72,457 | 55.2 |
| 1 | 15,602 | 11.9 |
| 2 | 7,441 | 5.7 |
| 3 | 4,465 | 3.4 |
| 4 | 2,864 | 2.2 |
| 5 | 2,052 | 1.6 |
| 6 | 1,478 | 1.1 |
| 7 | 1,273 | 1.0 |
| 8 | 1,035 | 0.8 |
| 9 | 795 | 0.6 |
| 10 | 749 | 0.6 |
| >10 | 5,279 | 4.0 |
| Unknown | 15,688 | 12.0 |

Supplementary Table S2. Patient characteristics

| Characteristics | No. | % |
| --- | --- | --- |
| Age (49.3 ± 10.6) |  |  |
| <35 | 7608 | 5.8 |
| 35≤, <50 | 62047 | 47.3 |
| 50≤ | 61523 | 46.9 |
| Operation Method |  |  |
| Mastectomy | 63083 | 48.1 |
| BCS | 66280 | 50.5 |
| Biopsy | 237 | 0.2 |
| Unknown | 1578 | 1.2 |
| T stage |  |  |
| T1 | 72596 | 55.3 |
| T2 | 50911 | 38.8 |
| T3 | 6169 | 4.7 |
| T4 | 1385 | 1.1 |
| Unknown | 117 | 0.1 |
| N stage |  |  |
| N0 | 81827 | 62.4 |
| N1 | 36253 | 27.6 |
| N2 | 8888 | 6.8 |
| N3 | 3997 | 3.0 |
| Unknown | 213 | 0.2 |
| Stage |  |  |
| I | 55308 | 42.2 |
| II | 57955 | 44.2 |
| III | 17915 | 13.7 |
| Histology grade |  |  |
| G1 | 17428 | 13.3 |
| G2 | 47325 | 36.1 |
| G3 | 37040 | 28.2 |
| Unknown | 29385 | 22.4 |
| Nuclear grade |  |  |
| G1 | 9899 | 7.5 |
| G2 | 44379 | 33.8 |
| G3 | 35146 | 26.8 |
| Unknown | 41754 | 31.9 |
| Lymphovascular invasion |  |  |
| Yes | 28644 | 21.8 |
| No | 61685 | 47.0 |
| Unknown | 40849 | 31.1 |
| Estrogen receptor |  |  |
| Positive | 74623 | 56.9 |
| Negative | 37755 | 28.8 |
| Unknown | 18800 | 14.3 |
| Progesterone receptor |  |  |
| Positive | 64838 | 49.4 |
| Negative | 46187 | 35.2 |
| Unknown | 20153 | 15.4 |
| HER2 |  |  |
| Positive | 21247 | 16.2 |
| Negative | 81090 | 61.8 |
| Unknown | 28841 | 22.0 |
| Radiation therapy |  |  |
| Yes | 64476 | 49.2 |
| No | 37826 | 28.8 |
| Unknown | 28876 | 22.0 |
| Chemotherapy |  |  |
| Yes | 78806 | 60.1 |
| No | 28147 | 21.5 |
| Unknown | 24225 | 18.4 |
| Endocrine therapy^a^ |  |  |
| Yes | 69643 | 53.1 |
| No | 29873 | 22.8 |
| Unknown | 31662 | 24.1 |
| Subtype |  |  |
| HR+/HER2- | 61137 | 46.6 |
| HR+/HER2+ | 10700 | 8.2 |
| HR-/HER2+ | 10349 | 7.9 |
| HR-/HER2- | 18829 | 14.3 |
| Unknown | 30163 | 23.0 |

Abbreviations: BCS, breast conserving surgery; HR, hormone receptor; HER2, human epidermal growth factor receptor 2

^a^Endocrine therapy was only applied to HR+ tumours.

Supplementary Table S3. Cox Proportional Hazard Regression Model for subtypes (univariate)

|  | All patients | | | HR+/HER2- | | | HR+/HER2+ | | | HR-/HER2+ | | | HR-/HER2- | | |
| --- | --- | --- | --- | --- | --- | --- | --- | --- | --- | --- | --- | --- | --- | --- | --- |
|  | HR | 95% CI | P | HR | 95% CI | *P* | HR | 95% CI | *P* | HR | 95% CI | *P* | HR | 95% CI | *P* |
| Age | 1.025 | 1.023-1.028 | <0.001 | 1.036 | 1.032-1.040 | <0.001 | 1.012 | 1.004-1.021 | 0.003 | 1.012 | 1.004-1.021 | 0.003 | 1.036 | 1.032-1.040 | <0.001 |
| Mastectomy | 1 |  |  | 1 |  |  | 1 |  |  | 1 |  |  | 1 |  |  |
| BCS | 0.411 | 0.385-0.428 | <0.001 | 0.402 | 0.374-0.432 | <0.001 | 0.456 | 0.390-0.532 | <0.001 | 0.456 | 0.390-0.532 | <0.001 | 0.402 | 0.374-0.432 | <0.001 |
| T stage |  |  |  |  |  |  |  |  |  |  |  |  |  |  |  |
| T1 | 1 |  | <0.001 | 1 |  | <0.001 | 1 |  | <0.001 | 1 |  | <0.001 | 1 |  | <0.001 |
| T2 | 2.150 | 2.069-2.234 | <0.001 | 2.338 | 2.174-2.513 | <0.001 | 2.020 | 1.749-2.293 | <0.001 | 2.020 | 1.749-2.293 | <0.001 | 2.338 | 2.174-2.513 | <0.001 |
| T3 | 4.271 | 4.034-4.522 | <0.001 | 5.071 | 4.514-5.695 | <0.001 | 3.323 | 2.669-4.183 | <0.001 | 3.323 | 2.669-4.183 | <0.001 | 5.071 | 4.514-5.695 | <0.001 |
| T4 | 9.481 | 8.741-10.285 | <0.001 | 12.067 | 10.199-14.276 | <0.001 | 7.303 | 5.506-10.198 | <0.001 | 7.303 | 5.506-10.198 | <0.001 | 12.067 | 10.199-14.276 | <0.001 |
| N stage |  |  |  |  |  |  |  |  |  |  |  |  |  |  |  |
| N0 | 1 |  | <0.001 | 1 |  | <0.001 | 1 |  | <0.001 | 1 |  | <0.001 | 1 |  | <0.001 |
| N1 | 2.287 | 2.198-2.380 | <0.001 | 2.158 | 1.992-2.337 | <0.001 | 2.372 | 2.027-2.687 | <0.001 | 2.372 | 2.027-2.687 | <0.001 | 2.158 | 1.992-2.337 | <0.001 |
| N2 | 4.285 | 4.079-4.502 | <0.001 | 4.535 | 4.123-4.988 | <0.001 | 4.077 | 3.392-4.900 | <0.001 | 4.077 | 3.392-4.900 | <0.001 | 4.535 | 4.123-4.988 | <0.001 |
| N3 | 6.399 | 6.023-6.799 | <0.001 | 7.766 | 6.994-8.622 | <0.001 | 6.270 | 5.040-7.801 | <0.001 | 6.270 | 5.040-7.801 | <0.001 | 7.766 | 6.994-8.622 | <0.001 |
| Histology grade |  |  |  |  |  |  |  |  |  |  |  |  |  |  |  |
| G1 | 1 |  | <0.001 | 1 |  | <0.001 | 1 |  | <0.001 | 1 |  | <0.001 | 1 |  | <0.001 |
| G2 | 2.058 | 1.899-2.231 | <0.001 | 1.917 | 1.709-2.150 | <0.001 | 1.708 | 1.312-2.335 | <0.001 | 1.708 | 1.312-2.335 | <0.001 | 1.917 | 1.709-2.150 | <0.001 |
| G3 | 3.080 | 2.843-3.336 | <0.001 | 3.339 | 2.966-3.760 | <0.001 | 2.219 | 1.626-2.905 | <0.001 | 2.219 | 1.626-2.905 | <0.001 | 3.339 | 2.966-3.760 | <0.001 |
| Lymphovascular invasion | 2.462 | 2.349-2.580 | <0.001 | 2.259 | 2.093-2.437 | <0.001 | 2.546 | 2.171-2.977 | <0.001 | 2.546 | 2.171-2.977 | <0.001 | 2.259 | 2.093-2.437 | <0.001 |
| Radiation therapy | 0.844 | 0.810-0.880 | <0.001 | 0.778 | 0.736-0.847 | <0.001 | 0.686 | 0.576-0.819 | <0.001 | 0.686 | 0.576-0.819 | <0.001 | 0.778 | 0.736-0.847 | <0.001 |
| Chemotherapy | 1.602 | 1.517-1.691 | <0.001 | 1.549 | 1.420-1.691 | <0.001 | 1.543 | 1.276-1.876 | <0.001 | 1.543 | 1.276-1.876 | <0.001 | 1.549 | 1.420-1.691 | <0.001 |
| Endocrine therapy^a^ | 0.622 | 0.596-0.649 | <0.001 | 0.599 | 0.533-0.672 | <0.001 | 0.604 | 0.486-0.754 | <0.001 | N.A. |  |  | N.A. |  |  |

Abbreviations: BCS, breast conserving surgery; HR, hormone receptor; HER2, human epidermal growth factor receptor 2; N.A., not applicable.

This analysis is for the whole study group of 131,178 patients.

^a^Endocrine therapy was only applied to HR+ tumours.

Supplementary Table S4. Cox Proportional Hazard Regression Model for subtypes (multivariate)

|  | All patients | | | HR+/HER2- | | | HR+/HER2+ | | | HR-/HER2+ | | | HR-/HER2- | | |
| --- | --- | --- | --- | --- | --- | --- | --- | --- | --- | --- | --- | --- | --- | --- | --- |
|  | HR | 95% CI | P | HR | 95% CI | *P* | HR | 95% CI | *P* | HR | 95% CI | *P* | HR | 95% CI | *P* |
| Age | 1.025 | 1.023-1.028 | <0.001 | 1.035 | 1.031-1.039 | <0.001 | 1.013 | 1.005-1.022 | 0.001 | 1.012 | 1.008-1.017 | <0.001 | 1.013 | 1.005-1.021 | 0.001 |
| Mastectomy | 1 |  |  | 1 |  |  | 1 |  |  | 1 |  |  | 1 |  |  |
| BCS | 0.612 | 0.586-0.639 | <0.001 | 0.668 | 0.608-0.735 | <0.001 | 0.640 | 0.544-0.752 | 0.012 | 0.762 | 0.653-0.888 | 0.001 | 0.597 | 0.466-0.765 | <0.001 |
| T stage |  |  |  |  |  |  |  |  |  |  |  |  |  |  |  |
| T1 | 1 |  | <0.001 | 1 |  | <0.001 | 1 |  | <0.001 | 1 |  | <0.001 | 1 |  | <0.001 |
| T2 | 1.452 | 1.392-1.514 | <0.001 | 1.508 | 1.390-1.635 | <0.001 | 1.426 | 1.219-1.668 | <0.001 | 1.420 | 1.246-1.619 | <0.001 | 1.479 | 1.200-1.792 | <0.001 |
| T3 | 2.064 | 1.938-2.199 | <0.001 | 2.172 | 1.906-2.476 | <0.001 | 1.596 | 1.243-2.050 | <0.001 | 2.773 | 2.277-3.377 | <0.001 | 2.002 | 1.485-2.697 | <0.001 |
| T4 | 3.687 | 3.371-4.033 | <0.001 | 4.236 | 3.532-5.080 | <0.001 | 3.246 | 2.317-4.547 | <0.001 | 4.436 | 3.360-5.857 | <0.001 | 4.025 | 2.756-5.877 | <0.001 |
| N stage |  |  |  |  |  |  |  |  |  |  |  |  |  |  |  |
| N0 | 1 |  | <0.001 | 1 |  | <0.001 | 1 |  | <0.001 | 1 |  | <0.001 | 1 |  | <0.001 |
| N1 | 1.921 | 1.840-2.006 | <0.001 | 1.824 | 1.665-1.997 | <0.001 | 1.952 | 1.639-2.326 | <0.001 | 1.617 | 1.405-1.860 | <0.001 | 2.556 | 2.035-3.210 | <0.001 |
| N2 | 2.887 | 2.731-3.051 | <0.001 | 3.095 | 2.756-3.476 | <0.001 | 2.870 | 2.320-3.550 | <0.001 | 2.651 | 2.211-3.179 | <0.001 | 3.402 | 2.565-4.512 | <0.001 |
| N3 | 3.892 | 3.636-4.167 | <0.001 | 4.576 | 4.013-5.217 | <0.001 | 4.157 | 3.235-5.342 | <0.001 | 3.731 | 3.066-4.639 | <0.001 | 4.907 | 3.671-6.559 | <0.001 |
| Histology grade |  |  |  |  |  |  |  |  |  |  |  |  |  |  |  |
| G1 | 1 |  | <0.001 | 1 |  | <0.001 | 1 |  | 0.040 | 1 |  | <0.001 | 1 |  | <0.001 |
| G2 | 1.549 | 1.427-1.683 | <0.001 | 1.519 | 1.351-1.708 | <0.001 | 1.548 | 1.046-2.356 | 0.008 | 0.971 | 0.713-1.323 | 0.852 | 0.944 | 0.566-1.574 | 0.826 |
| G3 | 1.946 | 1.790-2.115 | <0.001 | 2.199 | 1.943-2.490 | <0.001 | 1.819 | 1.342-2.608 | 0.004 | 1.132 | 0.839-1.528 | 0.417 | 1.081 | 0.654-1.789 | 0.761 |
| Lymphovascular invasion | 1.313 | 1.247-1.382 | <0.001 | 1.143 | 1.049-1.245 | 0.002 | 1.507 | 1.271-1.786 | 0.002 | 1.548 | 1.368-1.752 | <0.001 | 1.476 | 1.229-1.773 | <0.001 |
| Radiation therapy | 0.872 | 0.804-0.946 | 0.001 | 0.871 | 0.794-0.956 | 0.004 | 0.788 | 0.619-1.003 | 0.053 | 0.949 | 0.819-1.100 | 0.489 | 0.795 | 0.568-0.904 | <0.001 |
| Chemotherapy | 0.711 | 0.670-0.755 | <0.001 | 0.669 | 0.605-0.740 | <0.001 | 0.726 | 0.423-0.853 | 0.004 | 0.761 | 0.615-0.941 | 0.012 | 0.543 | 0.402-0.734 | <0.001 |
| Endocrine therapy^a^ | 0.668 | 0.639-0.698 | <0.001 | 0.697 | 0.620-0.785 | <0.001 | 0.615 | 0.497-0.770 | <0.001 | N.A. |  |  | N.A. |  |  |

Abbreviations: BCS, breast conserving surgery; HR, hormone receptor; HER2, human epidermal growth factor receptor 2; N.A., not applicable.

This analysis is for the whole study group of 131,178 patients.

^a^Endocrine therapy was only applied to HR+ tumours.

Supplementary Table S5. Adjusted risks of death according to different T and N stages.

|  | HR+/HER2- | | HR+/HER2+ | | HR-/HER2+ | | HR-/HER2- | |
| --- | --- | --- | --- | --- | --- | --- | --- | --- |
|  | HR | 95% CI | HR | 95% CI | HR | 95% CI | HR | 95% CI |
| T stages |  |  |  |  |  |  |  |  |
| T1 | ref |  | 1.432 | 1.238-1.657 | 1.375 | 1.181-1.600 | 1.936 | 1.729-2.167 |
| T2 | 1.646 | 1.505-1.801 | 2.179 | 1.915-2.480 | 2.347 | 2.062-2.627 | 2.836 | 2.564-3.137 |
| T3 | 2.839 | 2.344-3.439 | 3.019 | 2.150-4.241 | 3.083 | 2.247-4.230 | 5.304 | 4.334-6.491 |
| T4 | 4.390 | 3.245-5.940 | 5.339 | 3.015-9.457 | 9.526 | 6.083-14.918 | 6.736 | 4.463-10.165 |
| N stages |  |  |  |  |  |  |  |  |
| N0 | ref |  | 1.292 | 1.126-1.481 | 1.149 | 0.999-1.322 | 1.808 | 1.639-1.994 |
| N1 | 1.689 | 1.536-1.857 | 2.455 | 2.134-2.824 | 2.882 | 2.501-3.322 | 3.100 | 2.768-3.470 |
| N2 | 2.815 | 2.461-3.219 | 3.316 | 2.627-4.185 | 4.241 | 3.412-5.271 | 4.618 | 3.888-5.485 |
| N3 | 3.040 | 1.901-4.862 | 6.158 | 2.550-14.869 | 3.476 | 1.647-7.336 | 4.580 | 2.688-7.805 |

This analysis is for the whole study group of 131,178 patients.
